# Adaptive Traits and Molecular Mechanisms of *Rhododendron* Species in Changbai Mountains’ Alpine Tundra: A Phenotype–Transcriptome Study

**DOI:** 10.3390/plants14233602

**Published:** 2025-11-26

**Authors:** Zhongzan Yang, Jian You, Jiangnan Li, Wei Zhao, Ming Xing, Yuqiao Gong, Xia Chen

**Affiliations:** National & Local United Engineering Laboratory for Chinese Herbal Medicine Breeding and Cultivation, School of Life Sciences, Jilin University, Qianjin Avenue 2699, Changchun 130012, China; yangzhongzan902@163.com (Z.Y.);

**Keywords:** *Rhododendron*, alpine tundra, Changbai Mountains, phenotypic plastic adaptation, morphology

## Abstract

Alpine tundra’s harsh conditions challenge plants, but *Rhododendron*’s adaptive mechanisms remain unclear. This study explored phenotypic/transcriptomic adaptations of three *Rhododendron* species (*R. aureum*, *R. lapponicum*, *R. redowskianum*) in Changbai Mountains’ tundra vs. timberline. Mature leaves were sampled for leaf length and leaf width measurement and RNA-seq. Results showed leaf width (not leaf length uniformly) reduced in tundra across all species. RNA-seq identified 2399–5716 DEGs per species; plant dwarfism DEGs (e.g., DELLA, EDS1) were up-regulated. Shared DEGs were enriched in carbon/nitrogen metabolism and stress response; IPUT1 (DUH022406.1) and PGT1 (DUH001929.1) were consistently down-regulated (linked to dwarfism). Species-specific responses included *R. aureum*’s light adaptation, *R. lapponicum*’s freezing/hypoxia response, and *R. redowskianum*’s sugar/UV/microbial regulation. *Rhododendron* adapts to tundra via leaf width adjustment, metabolic optimization, and IPUT1/PGT1-mediated dwarfism, with conserved core mechanisms and species specialization, supporting climate change response predictions and conservation.

## 1. Introduction

*Rhododendron* represents the largest genus of woody plants in the Northern Hemisphere and constitutes a significant component of montane ecosystems [1]. This genus, belonging to the Ericaceae family, boasts remarkable diversity, with over 1000 species described, distributed across continents from Asia to North America and Europe [2]. Their ecological roles are multifaceted, ranging from foundational species in forest understories to dominant shrubs in alpine scrublands [3]. Due to its robust ecological adaptability, rapid growth, and reproductive capabilities, *Rhododendron* can function as a pioneering species capable of facilitating secondary succession across diverse environments [4,5]. For instance, following disturbances such as landslides or glacial retreat, *Rhododendron* often forms dense thickets that stabilize soils and create microclimates favorable for the establishment of other plant species, thereby initiating successional sequences [6]. Previous research has indicated that *Rhododendron* species exhibit substantial climatic adaptability, including cold tolerance and wind resistance, as well as soil adaptability, such as tolerance to infertile and acidic soils [7,8,9,10,11]. These attributes, along with their diversified reproductive strategies, are considered primary factors contributing to their survival in tundra ecosystems. They are of great significance for maintaining the ecological balance of the alpine tundra and for ecological conservation.

The tundra, representing the upper limit of terrestrial environments where higher plants can grow, is characterized by its harsh conditions. *Rhododendron* species are well-adapted to colonize these tundra environments [5,12]. However, global climate change has led to the degradation of *Rhododendron* populations in high mountain areas [13,14,15]. Studies have shown that under climate change, some species of the *Rhododendron* genus exhibit range expansion, while others experience range contraction, and some of the latter even face the risk of extinction [16,17]. The Changbai Mountain tundra, a notable example of China’s alpine tundra, supports a diverse array of higher plants, including *Rhododendron aureum* Franch. (*R. aureum*), *Rhododendron lapponicum* (L.) Wahlenb. (*R. lapponicum*), and *Rhododendron redowskianum* Rehder & E. H. Wilson (*R. redowskianum*) within the genus *Rhododendron* [18,19]. On the alpine tundra of Changbai Mountain, *R. aureum*, *R. lapponicum*, and *R. redowskianum* are dominant dwarf shrubs [18]. *R. aureum* is the most dominant dwarf shrub [18,20]. Under climate change, *R. redowskianum* has shown a declining distribution trend [12,21], while *R. lapponicum* may exhibit an increasing distribution trend [21]. In general, the dominance of dwarf shrubs has declined compared with tundra herbs [20]. These three species co-occur but may occupy slightly different microhabitats within the tundra matrix. *R. aureum*, *R. lapponicum* and *R. redowskianum* are found in the mountain regions of Asia, Europe and even North America [18,22,23,24,25]. This wide distribution makes them excellent models for studying convergent and divergent adaptation mechanisms across geographical scales.

Plants frequently exhibit adaptations to alpine tundra environments through reduced stature. While the phenotypic manifestations of alpine adaptation, such as dwarfism, are well-documented [26], the genetic and molecular pathways orchestrating these changes—including the roles of specific hormones like brassinosteroids and key transcription factors—are still not being deciphered [27]. However, the underlying molecular mechanisms remain insufficiently understood. While previous studies have investigated the physiological responses of *Rhododendron* to individual stressors (e.g., cold tolerance in *Rhododendron* [28] and UV-B resistance in *Rhododendron chrysanthum* [29]), few have integrated phenotypic and transcriptomic data to explore comprehensive adaptation strategies in alpine tundra environments. Elucidating these mechanisms is crucial for comprehending plant migration in response to climate change, particularly from their native habitats to increasingly warmer regions [12,30]. Understanding the genetic basis of adaptation allows for better predictions of species’ range shifts, identifies populations at risk, and informs conservation strategies aimed at preserving alpine biodiversity in a warming world.

Under global climate change, the *Rhododendron* genus plays a crucial role in maintaining the stability and supporting the restoration of harsh alpine ecosystems [1,31]. To our knowledge, there has been no specific molecular biological transcriptomic research on the *Rhododendron* genus in alpine tundra previously. Against this backdrop, the present study aimed to (1) characterize the phenotypic responses (specifically leaf morphology) of three dominant *Rhododendron* species (*R. aureum*, *R. lapponicum*, *R. redowskianum*) to alpine tundra versus timberline habitats in Changbai Mountains; (2) identify differentially expressed genes (DEGs) associated with tundra adaptation using RNA sequencing (RNA-seq); (3) elucidate shared and species-specific molecular mechanisms underlying adaptation, with a focus on stress response and metabolic pathways; and (4) link transcriptomic changes to phenotypic traits (e.g., dwarfism) to provide a holistic understanding of adaptive plasticity in *Rhododendron*. We hypothesize that a combination of conserved, genus-wide molecular responses and species-specific genetic adjustments underlies the successful colonization of the alpine tundra by these *Rhododendron* species. This study aims to investigate the factors driving the phenotypic plastic adaptation of the genus *Rhododendron* to alpine tundra conditions and to identify the specific molecular mechanisms facilitating this adaptation.

## 2. Results

### 2.1. Leaf Phenotypic Responses of Rhododendron spp.

**Figure 1:**
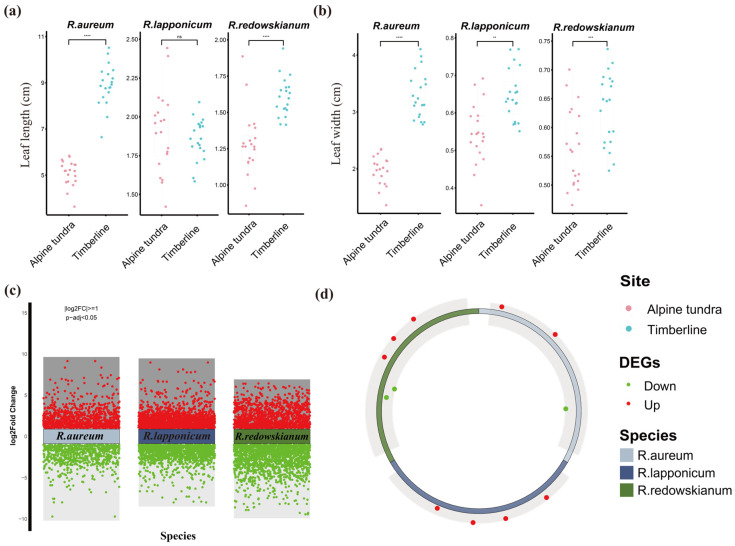
Indicators of leaf morphology and DEGs of *Rhododendron* spp. in alpine tundra and timberline. Leaf length (**a**) and leaf width (**b**) of *Rhododendron* spp. (*n* = 20). The Wilcoxon rank-sum test was used to evaluate the significance of differences (**** *p* < 0.0001 (extremely highly significant); *** *p* < 0.001 (extremely significant); ** *p* < 0.01 (highly significant); NS, Not Significant, *p* ≥ 0.05), and the tops and bottoms of the boxes show the 75th and 25th percentiles, respectively. (**c**) DEGs of *Rhododendron* spp. (**d**) DEGs associated with leaf development.

Among the three *Rhododendron* species present in alpine tundra and timberline environments, leaf length was compared between the two habitats (alpine tundra vs. timberline). Significant differences (*p* < 0.05) were observed for *R. aureum* and *R. redowskianum*, while no such difference was detected for *R. lapponicum*. This suggests that *R. lapponicum* may possess a more canalized development for leaf length, making it less plastic in response to the environmental gradient between timberline and tundra, or that its specific adaptive strategy does not involve modifying this particular trait. In contrast, leaf width exhibited significant differences (*p* < 0.05) between the habitats for all three species (Figure 1a,b; Figure S1; Table S1). The consistent reduction in leaf width observed in the tundra across all species points to a convergent adaptive response.

### 2.2. Characteristics of Differentially Expressed Genes (DEGs) in Rhododendron spp.

To further understand the causes of morphological changes in the leaves of *Rhododendron* spp., RNA-seq sequencing was conducted, utilizing timberline as a control. The timberline site, being less extreme than the tundra but sharing similar flora, serves as an ideal reference to isolate gene expression changes specifically associated with adaptation to the harsher tundra conditions, rather than general altitudinal or species-specific effects [32]. The statistical parameters analyzed are detailed in Table S2. A substantial number of DEGs (|log2FC| ≥ 1 and *p*-adj < 0.05) were identified for each species, with counts ranging from 2399 to 5716 (Figure 1c, Table S3). The variation in the total number of DEGs among species indicates differing magnitudes of transcriptional reprogramming required for acclimation, potentially reflecting their varying degrees of pre-adaptation or their distinct ecological strategies. Most of the DEGs associated with leaf development were found to appear up-regulated (Figure 1d), and these up-regulated differential genes were associated with transcription factors and disease resistance proteins, such as DELLA and EDS1 (Table S4). DELLA proteins are key negative regulators of gibberellin signaling, and their accumulation is known to restrain growth, promoting a dwarfed phenotype [27]. EDS1 is a central component of plant innate immunity, involved in salicylic acid signaling and defense against biotrophic pathogens [33].

### 2.3. Molecular Biological Responses of Rhododendron spp. to Alpine Tundra

**Figure 2:**
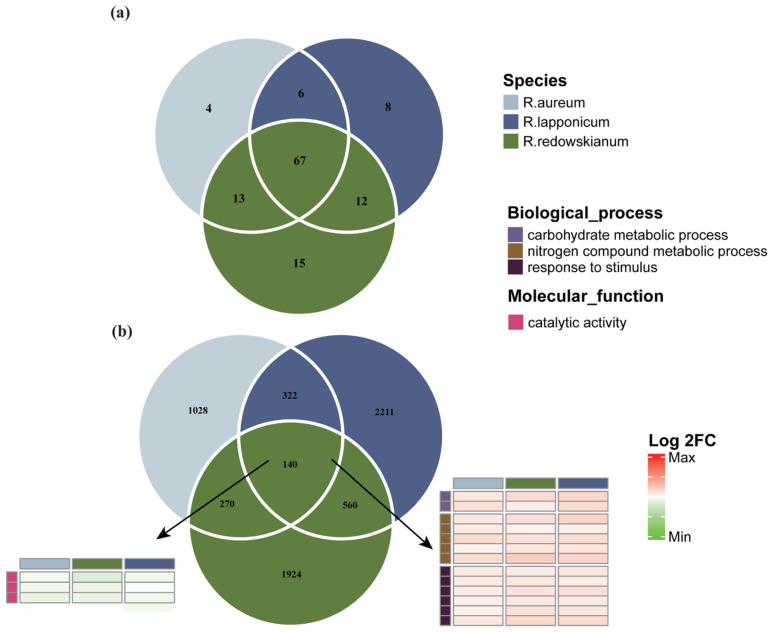
DEGs and their annotations. (**a**) Venn diagram representing DEGs annotated to environmentally generated stimuli. (**b**) Venn plot representing DEGs number and some of their annotations.

The annotation of these DEGs indicated a significant number of genes involved in responses to environmental stimuli in *Rhododendron* spp. Venn diagrams illustrate the types of stimuli managed within the biological processes of various species (Figure 2a). This analysis effectively partitions the “stressome” of the *Rhododendron* community, revealing the shared and unique challenges perceived at the molecular level. The shared stimuli, as depicted in Table S5, reflect the adaptation of *Rhododendron* spp. to the alpine tundra. These stimuli encompass responses to temperature fluctuations, injury, ultraviolet radiation, and osmotic pressure, among others. This core set of shared responses defines the common molecular signature of alpine tundra adaptation for these species, highlighting the multi-stress nature of this environment where plants must simultaneously cope with a barrage of abiotic pressures.

**Figure 3:**
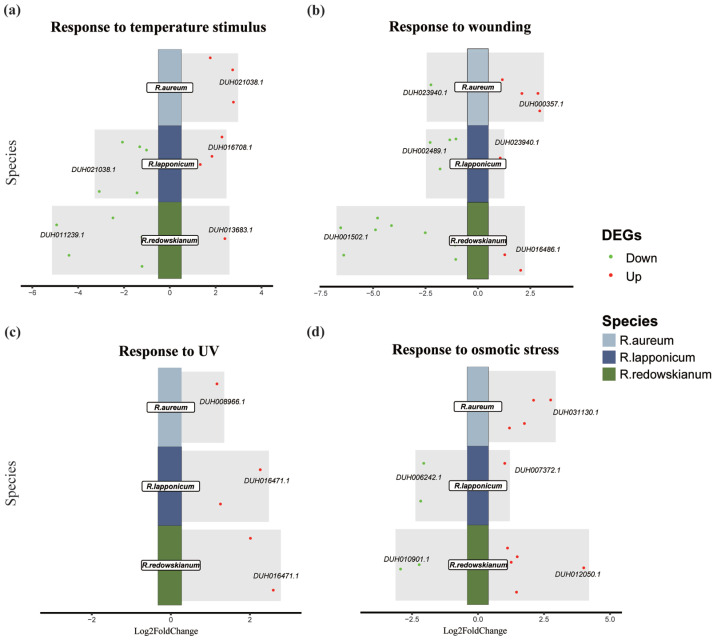
DEGs of *Rhododendron* spp. in response to temperature stimuli (**a**), injury (**b**), UV light (**c**), and osmotic stress stimuli (**d**). Gene_id represents |log2FC| maximal DEG.

In response to temperature stimulus (Figure 3a), wounding (Figure 3b), UV stimulus (Figure 3c) and osmotic stress stimulus (Figure 3d), the three species did not exhibit a uniform up-regulation of proteins in reaction to identical stimuli (Tables S6–S9). This observation underscores the distinct mechanisms by which different species respond to the same environmental challenges. This species-specificity in molecular responses likely stems from their distinct evolutionary histories, genetic backgrounds, and possibly fine-scale differences in their microhabitat preferences within the tundra [34,35]. The stimuli to which *R. aureum* exclusively responded were associated with light (Table S10), resulting in the down-regulation of DEGs (Table S11). The down-regulation of light-responsive genes in *R. aureum* could be an adaptation to potentially damaging high light intensity (including UV) in the tundra, perhaps involving the down-regulation of components of photosynthesis light-harvesting complexes to prevent photo-oxidation or adjustments in photoperiod sensing mechanisms. In contrast, *R. lapponicum* uniquely responded to stimuli related to freezing temperatures or reduced oxygen levels (Tables S12 and S13). This specific focus suggests that *R. lapponicum* is particularly sensitive or specially adapted to the most extreme low-temperature and potentially anoxic conditions. *R. redowskianum* exhibited responses to stimuli involving self-produced sugars, UV light, and microorganisms (Tables S14 and S15). The response to sucrose or other sugars might indicate a role for sugar signaling in coordinating its stress responses. The specific UV response could involve unique flavonoid biosynthesis genes for screening UV-B radiation. The microbe-related responses hint at species-specific interactions with the rhizosphere or phyllosphere microbiome, which could be crucial for nutrient acquisition or disease resistance in its particular niche.

**Figure 4:**
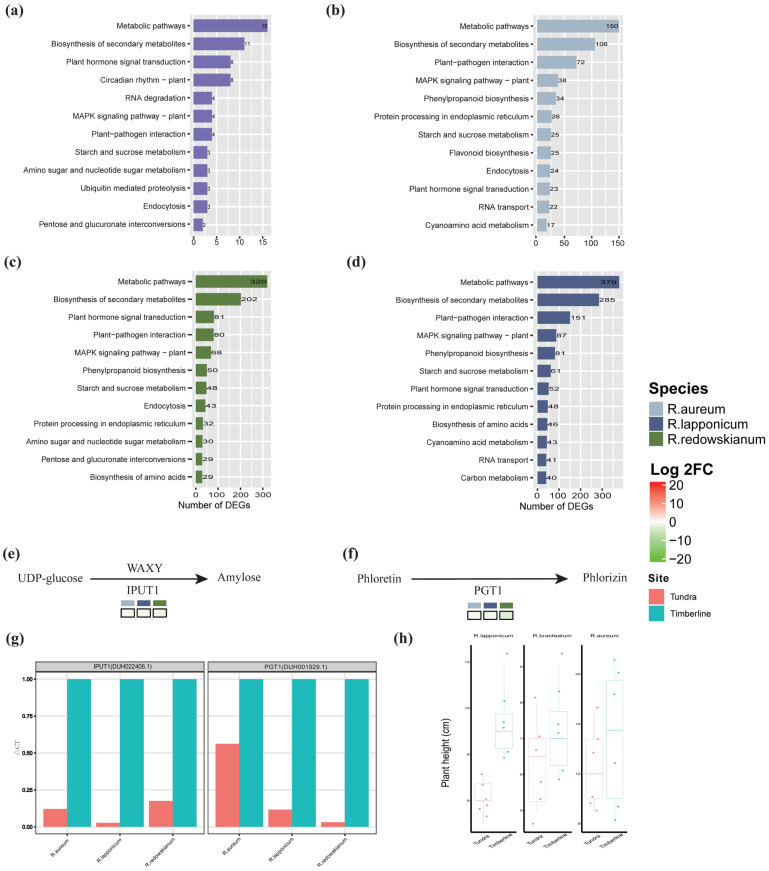
Top 12 KEGG annotations for DEGs common to *Rhododendron* spp. (**a**) and unique to *R. aureum* (**b**), *R. lapponicum* (**c**) and *R. redowskianum* (**d**). Down-regulated DEGs of *Rhododendron* spp. annotated to IPUT1 (**e**) and PGT1 (**f**). (**g**) RT-qPCR validation. (**h**) The plant height of *Rhododendron* refers to the vertical height from the top of the plant to the ground.

Venn diagram analysis of DEGs in the genus *Rhododendron* (Figure 2b) identified common up-regulation in shared DEGs associated with carbon metabolism, nitrogen compound metabolism, and response to stimuli (Table S16). These findings are indicative of the adaptive response of *Rhododendron* species to the tundra environment. Conversely, the commonly down-regulated DEGs were related to enzyme activities (Figure 2b, Table S17). This general down-regulation of various enzyme activities could reflect an overall slowing of metabolic processes not immediately critical for survival under stress, a conservation of resources, or a re-prioritization of cellular activities [36,37]. It might also be a consequence of the growth reduction, as many metabolic enzymes are correlated with growth rate. The KEGG annotation of DEGs unique to and shared by various species of the genus *Rhododendron* (Figure 4a–d) indicated that, in addition to the well-established pathways such as MAPK signaling cascades, plant–pathogen interactions, and hormone signaling pathways, there was a significant representation of DEGs associated with “starch and sucrose metabolism (ko00500)”. The prominence of starch and sucrose metabolism is highly significant [38]. Carbohydrate metabolism is central to plant energy balance, carbon storage, and signaling. Changes in sugar composition can also act as osmoprotectants against freezing and desiccation [39]. Among these findings, a consistent down-regulation of the DEG DUH022406.1 was observed across *Rhododendron* species on IPUT1 (Inositol Phosphorylceramide Glucuronosyltransferase 1) (Figure 4e, Table S17), correlating with a reduction in straight-chain starch production. Additionally, PGT1 (phlorizin synthase) exhibited a uniform down-regulation of the common DEG DUH001929.1 (Figure 4f, Table S17), which is associated with a decreased production of rhizopiridin. The expression levels of these two DEGs were validated by RT-qPCR (Figure 4g).

## 3. Discussion

*Rhododendron* species exhibit significant phenotypic plastic adaptations to alpine tundra environments. Our study moves beyond correlative observations and provides a molecular-genetic foundation for understanding this plasticity, identifying specific genes and pathways involved, and elucidating the common responses of three *Rhododendron* genera to alpine tundra conditions, focusing on their response to the environment, particularly carbon and nitrogen metabolism. By integrating phenotypic data with transcriptomic profiles, we bridge the gap between form and function, offering a more holistic view of adaptation. These metabolic pathways, including sucrose and starch metabolism, are crucial for survival in alpine tundra. Our study further adds to the understanding of the adaptation of *Rhododendron* spp. to alpine tundra environments. It does so by revealing both a common adaptive core and a layer of species-specific specialization, integrated community-level adaptation through complementary strategies.

### 3.1. Causes of Changes in Leaf Morphology

In this study, we conducted a comprehensive investigation into various characteristics of three *Rhododendron* species inhabiting alpine tundra and timberline environments, with a particular focus on changes in leaf morphology and adaptive molecular mechanisms. Regarding leaf morphology, prior research has indicated that altitude is the primary determinant of leaf shape, rather than leaf age [40]. Altitude acts as a complex proxy for multiple covarying environmental factors, including temperature, radiation, wind, and atmospheric pressure, all of which can influence leaf development [41,42]. Our findings corroborate this assertion and further elucidate that leaf width is the critical factor influencing leaf shape within the *Rhododendron* genus in alpine tundra ecosystems. Width reduction is a more universal response in these species than length reduction, possibly because it more directly mitigates mechanical stress from wind and improves thermal regulation [43]. A narrower leaf blade can reduce the boundary layer thickness, enhancing heat exchange and reducing water loss under the strong winds typical of alpine summits [44]. These findings suggest that leaf width is a primary factor contributing to the morphological variations in leaf blades of *Rhododendron* species transitioning from timberline to alpine tundra.

DEGs associated with leaf development (see Table S4) indicate that distinct *Rhododendron* species possess unique regulatory mechanisms for leaf development. Notably, one such mechanism involves the partial up-regulation of the “DELLA—EDS1 Module”, which plays a crucial role in mediating the balance between plant growth and defense. Through this negative feedback regulatory mechanism, or potentially through individual negative regulatory mechanisms specific to each species, precise control over the equilibrium between plant growth and defense can be attained [45]. In the context of the resource-limited tundra, investing heavily in defense (e.g., via EDS1) at the expense of growth (restrained by DELLA) is likely a beneficial strategy [45]. The dwarfed stature itself can be a form of morphological defense, reducing exposure to harsh elements. The co-regulation of these modules suggests an integrated molecular network that fine-tunes the plant’s resource allocation strategy in response to environmental severity. The precise regulatory mechanisms and the direct correlations with specific environmental factors require further investigation.

### 3.2. Adaptive Response of Rhododendron spp. in Alpine Tundra

At the level of gene expression, RNA-seq sequencing results revealed rich and complex patterns of adaptation. The transcriptome acts as a dynamic interface between the genome and the environment, and its remodeling in the tundra reflects the active reprogramming necessary for life at the edge. Different species showed differences in response to the same stimuli, such as temperature, injury, UV stimulation and osmotic stress stimulation, without common up-regulated proteins, suggesting that each species has developed respective coping strategies. This finding challenges a simplistic view of adaptation and emphasizes the importance of considering intra-generic diversity. Conservation strategies may need to be species-specific, as a threat affecting one species’ unique adaptive pathway might not impact another. This diversity may stem from differences in the genetic background of the species, their long evolutionary history, and the specific ecological niche they occupy in the alpine tundra ecosystem. Our study indicates that *Rhododendron* spp. in the alpine tundra are subjected to both high and low temperature stimuli. The tundra environment, while generally cold, can experience high solar irradiance, leading to significant leaf heating on sunny days, creating a scenario where plants must be prepared for both freezing and heat stress, sometimes within a 24 h cycle [32]. Investigating their responses to these thermal stimuli should be prioritized in research, as cold exposure can result in irreversible damage to the plants [46]. Understanding the mechanisms of recovery from both hot and cold stimuli is a critical area of research, given that responses to thermal stress vary among different species [47]. Additionally, plant damage in the alpine tundra may be exacerbated by mechanical damage, strong winds, and insect herbivory, further complicating their ability to cope with thermal stress [48].

In the alpine tundra, various *Rhododendron* species exhibit distinct responses, as detailed in Figure S3 (Table S10), Figure S4 (Table S12), and Figure S5 (Table S14). Specifically, the response of *R. lapponicum* to icing and hypoxia suggests a survival strategy under extreme low temperatures and oxygen deficiency, potentially involving the expression of antifreeze proteins or enhanced oxygen utilization efficiency. Conversely, the distinct responses of *R. redowskianum* to stimuli such as self-produced sugars, UV light, and microbial interactions may indicate unique regulatory mechanisms for energy reserves, UV damage resistance, and microbial interactions. Sugars can act as signaling molecules that integrate information about the plant’s carbon status and regulate stress-responsive genes [38]. Compared to the other two species, *R. aureum* exhibited superior adaptation to the alpine tundra environment and did not require additional up-regulation of DEGs for acclimatization, unlike other species within the genus *Rhododendron*. This could be interpreted as *R. aureum* being inherently pre-adapted or “hard-wired” for the tundra conditions. Its constitutive gene expression levels might already be sufficient, requiring less plastic adjustment. Alternatively, its key adaptations might operate at the post-transcriptional, translational, or metabolic level, not captured by RNA-seq. This inherent adaptability may account for the robust presence of *R. aureum* in the alpine tundra of the Changbai Mountains, even amidst climate change [12,20]. These findings underscore the variability in the responses of different *Rhododendron* species to the alpine tundra environment. This functional diversity at the molecular level likely contributes to the stable coexistence of these species by reducing direct competition and allowing them to exploit slightly different aspects of the tundra environment.

### 3.3. Common Adaptive Mechanisms of Rhododendron spp.

A comprehensive analysis of the shared DEGs demonstrated that the co-up-regulated genes are intricately associated with carbon metabolism, nitrogen compound metabolism, and response to stimuli. This constitutes the “core transcriptomic response” to the alpine tundra, a set of fundamental processes that are non-negotiable for survival there, regardless of species-specific nuances. This observation strongly suggests that these fundamental metabolic processes and stress responses are pivotal in facilitating the adaptation of *Rhododendron* spp. to the challenging conditions of the alpine tundra. The regulation of carbon and nitrogen compound metabolism enhances the efficiency of energy and material utilization in plants, while effective responses to stimuli facilitate rapid adaptation to environmental changes [49,50]. In essence, the common strategy is to become a metabolically efficient and highly responsive organism, capable of quickly turning limited resources into usable energy and activating defenses at a moment’s notice. Adjustments in this pathway are likely key to managing energy reserves over the long winter and mobilizing them rapidly at the start of the short growing season.

However, the observed co-down-regulation of certain genes associated with enzyme activities suggests that, in alpine tundra environments, the functionality of these enzymes may be more critical for survival and adaptation. Alternatively, their activities might be modulated through the suppression of environmental factors. In the KEGG annotation, a significant number of DEGs were implicated in the sucrose and starch metabolism pathways. Additionally, the consistent down-regulation of shared DEGs related to IPUT1 and PGT1 was closely linked to the reduction in straight-chain amylopectin and root bark glycoside production (Figure 4e,f). This discovery has profound implications for understanding the adaptive regulation of energy storage and secondary metabolite synthesis in *Rhododendron* spp. Decreased synthesis of straight-chain starch might represent an adaptive strategy to optimize energy storage forms in resource-limited environments [51,52]. Similarly, the reduced production of root bark glycosides could be linked to decreased metabolic costs or modifications in defense mechanisms.

A consistent down-regulation (Table S17) of genes shared by PGT1 (DUH001929.1) not only directly restricts pathogen proliferation and toxin production but also mediates the synthesis of salicylic acid (SA) and reactive oxygen species (ROS) [53]. This ultimately results in plants displaying suppressed growth and development alongside enhanced disease resistance [53]. This provides a potential molecular link between the observed dwarfism and the up-regulation of defense-related genes like EDS1 [33]. Down-regulating PGT1 might be part of a coordinated mechanism to simultaneously slow growth and prime defenses, a state potentially beneficial in a high-stress environment where resources are too scarce for rapid growth and the risk of infection at wound sites is high. Similarly, the down-regulation of IPUT1 (DUH022406.1) has been linked to plant dwarfism. IPUT1 is the essential enzyme that initiates the glycosylation of sphingolipids and is required for the synthesis of Glycosyl Inositol Phosphorylceramides (GIPCs). GIPCs are crucial for maintaining normal cellular membrane structure, facilitating signal transduction, and regulating programmed cell death in plants. Consequently, the loss of IPUT1 function directly disrupts GIPC biosynthesis, which in turn triggers severe developmental defects at both cellular and organismal levels, including growth retardation and reproductive failure [54]. This suggests that the down-regulation of both DUH001929.1 and DUH022406.1 could potentially contribute to the dwarfism observed in *Rhododendron* spp. in the alpine tundra environment (Figure 4h, plant height data from Yang et al. [32]). Thus, we propose a model where convergent phenotypic adaptation (dwarfism) is achieved, at least in part, through the convergent down-regulation of these two key genes, which likely operate in distinct molecular pathways (secondary metabolism/signaling and membrane lipid/signaling) that both ultimately impact growth regulation. Meanwhile, we will further refine variables in future indoor simulation experiments. Our goal is to accurately identify genes governing leaf width. This provides valuable guidance and insights for plant adaptation to harsh alpine environments. Injury, UV stimulation, and osmotic stress were identified via GO annotation. While this aligns with existing knowledge, no experimental validation was conducted. This will be verified in future studies.

## 4. Materials and Methods

### 4.1. Experimental Materials and RNA Extraction

We applied the principle of terrain similarity in Changbai Mountains’ alpine tundra (42.043–42.045° N, 128.073–128.079° E, 2250 ± 25 m) and timberline (42.054–42.056° N, 128.070–128.075° E, 2000 ± 20 m) to ensure the study’s comparability and reliability. On 28 July 2021, we collected over 18 mature, non-senescent leaves from each of three species: *R. aureum*, *R. lapponicum*, and *R. redowskianum* for measuring plant phenotypes. Climatic data on the sampling day at the study sites refers to Yang et al. [32]. Following the standardized protocols delineated by Harguindeguy et al. [55], leaves were sampled from a minimum of six individual plants per species, with at least 3 leaves collected from each plant. Specific sampling sites refer to Yang et al. [32]. This date was strategically chosen to coincide with the peak of the growing season, ensuring that sampled leaves were fully developed and metabolically active, thereby capturing gene expression profiles representative of acclimation to summer conditions in their respective habitats.

In this research, the objects of comparison for morphological changes are limited to two habitats: alpine tundra and timberline. Leaf width and length were measured, and leaves were obtained from no fewer than six sample individuals for storage in liquid nitrogen and subsequent experimental analysis. A total of 18 sequencing samples were collected, comprising three *Rhododendron* species (with three biological replicates each) from both tundra and timberline habitats. This balanced experimental design, featuring multiple species, two contrasting habitats, and biological replication, provides the statistical power necessary to discern both common and species-specific patterns of gene expression. Total RNA was extracted from leaves using a TRIzol kit (Invitrogen, Carlsbad, CA, USA), following the manufacturer’s protocol, which effectively isolates high-quality RNA by separating RNA from DNA and proteins through phase separation, and treated with 10 units of DNase I (Takara, Dalian, China) at 37 °C for 30 min to eliminate genomic DNA.

### 4.2. Sequencing Library Construction and Sequencing

The RNA quality was evaluated utilizing an Agilent 2100 Bioanalyzer (Agilent Techcologies, Santa Clara, CA, USA) in conjunction with the Agilent RNA 6000 Nano Kit (Agilent Technologies, Santa Clara, CA, USA). For cDNA library construction, 20 μg of RNA with an RNA Integrity Number (RIN) exceeding 8.0 was employed. Poly(A) mRNAs were enriched for each sample through Oligo(dT) selection. The mRNA was subsequently fragmented and reverse-transcribed using N6 random primers to produce double-stranded cDNA. This cDNA underwent end-repair and 3′ adenylation, followed by the ligation of adaptors to the adenylated cDNA fragments. The ligation products underwent purification and were subsequently subjected to multiple rounds of PCR amplification to enrich the cDNA template. Following amplification, the PCR products were denatured by heat, and the resulting single-stranded DNA was looped through splicing oligonucleotides and DNA ligase. A total of 18 libraries, with three replicates per sample, were sequenced for three *Rhododendron* species. Sequencing was performed in paired-end mode (2 × 100 bp) using the BGISEQ-500 sequencing platform (BGI-Shenzhen, China). RT-qPCR validation was performed following the protocol described by Yang et al. [32].

### 4.3. RNA-Seq Analysis and Data Processing

The raw sequencing data were filtered using SOAPnuke (v1.4.0) to obtain clean data [56]. Subsequently, Bowtie2 (v2.2.5) was employed for alignment. The clean data were then compared to the reference gene set, *Rhododendron ovatum* (GenBank: JADHZH000000000) [57,58]. The genome of *Rhododendron ovatum* has been successfully assembled, with its genomic integrity and continuity evaluated using the scaffold N50 and BUSCO. These two key genomic characteristics are higher than those of the previously assembled genomes from three other *Rhododendron* species [58]. Gene expression quantification was conducted using RSEM (v1.2.8) software [59]. Differential gene expression analysis was performed using DESeq2, with a significance threshold set at |log_2_(Fold Change)| ≥ 1 and Q value < 0.05 [60]. Enrichment analysis of DEGs for Gene Ontology (GO) and Kyoto Encyclopedia of Genes and Genomes (KEGG) pathways was conducted using the Phyper tool [61].

### 4.4. Data Collection and Analyses

Leaf length (LL) and leaf width (LW) were measured utilizing ImageJ (v1.53, National Institutes of Health, Bethesda, MD, USA). Statistical analyses were performed using R software (v4.3.1, http://www.r-project.org, accessed on 15 June 2024). The circular Volcano plot was generated employing the “scRNAtoolVis” package (v0.0.4) developed by Junjun Lab, accessible at https://github.com/junjunlab/scRNAtoolVIS, accessed on 15 June 2025. The Venn diagram was generated using the VennDiagram package (v1.7.3) in R (https://CRAN.R-project.org/package=2, accessed on 15 June 2025). Heat maps were constructed utilizing the ComplexHeatmap package (v2.12.1) in R (https://github.com/jokergoo/ComplexHeatmap, accessed on 20 June 2024). Bar plots were created with the ggplot2 package (v3.4.2) [62].

## 5. Conclusions

*Rhododendron* spp. exhibit several common adaptations within the alpine tundra ecosystem of Changbai Mountains. This study provides a multi-species, integrative perspective on alpine plant adaptation, combining ecology, morphology, and transcriptomics. Among these adaptations, leaf width emerges as a significant determinant of leaf morphology, and leaf development is a trade-off between growth and defense. These plants must contend with various abiotic stresses, including temperature fluctuations, physical injury, ultraviolet radiation, and osmotic stress. The transcriptomic data vividly captures this multi-stress reality, showing activation of diverse response pathways. The adaptive evolution to both high and low temperature stimuli is a critical factor enabling *Rhododendron* spp. to thrive in the alpine tundra. Thermal resilience appears to be a cornerstone of their success. Carbon and nitrogen metabolism are critical in the acclimatization processes, with particular emphasis on sucrose and starch metabolism. The down-regulation of IPUT1 (DUH022406.1) and PGT1 (DUH001929.1) may contribute to the observed dwarfism in *Rhododendron* spp. within alpine tundra environments. Key genes and phenotypic traits linked to cold adaptation were identified in this study. They provide theoretical references and technical support for breeding cold-resistant *Rhododendron* cultivars. Clarified adaptive strategies of the three *Rhododendron* species are reported. They offer scientific guidance for optimizing species selection in degraded alpine tundra restoration and also support habitat improvement in the ecological restoration of such ecosystems.

## Data Availability

Data are available on request from the authors. Transcriptome sequencing data have been submitted to the Short Read Archive (SRA) data library under accession number: PRJNA1153639.

## Supplementary Materials

The following supporting information can be downloaded at https://www.mdpi.com/article/10.3390/plants14233602/s1, Figure S1: Leaves of three *Rhododendron* species in the tundra and timberline: *R. aureum* (a), *R. lapponicum* (b), and *R. redowskianum* (c) inhabiting the tundra; and *R. aureum* (d), *R. lapponicum* (e), and *R. redowskianum* (f) growing at timberline; Figure S2: DEGs associated with leaf development annotated in *Rhododendron* spp.; Figure S3: Uniquely annotation to GO level 3 about stimuli in *R. aureum*. Name sizes are randomized, the same below; Figure S4: Uniquely annotation to GO level 3 about stimuli in *R. lapponicum*; Figure S5: Uniquely annotation to GO level 3 about stimuli in *R. redowskianum*. Table S1: Leaf length and width of *Rhododendron* species in alpine tundra and timberline; Table S2: RNA sequencing and assembly statistics for transcriptome data in *Rhododendron*; Table S3: The summary of DEGs of *Rhododendron* species; Table S4: DEGs associated with leaf development annotated in *Rhododendron* spp.; Table S5: Co-annotation of *Rhododendron* species’ DEGs to GO level 3 annotation of related stimuli; Table S6: *Rhododendron* spp. annotated to DEGs about temperature stimuli; Table S7: *Rhododendron* species annotated to DEGs about damage stimuli; Table S8: *Rhododendron* species annotated to DEGs about UV stimulation; Table S9: *Rhododendron* species annotated to DEGs about osmotic stress stimulation; Table S10: Uniquely annotation to GO level 3 about stimuli in *R. aureum*; Table S11: DEGs of *R. aureum* for response to far red light; Table S12: Uniquely annotation to GO level 3 about stimuli in *R. lapponicum*; Table S13: DEGs in *R. lapponicum* uniquely annotated to GO level 3 about freezing temperatures and reduced oxygen levels; Table S14: Uniquely annotation to GO level 3 about stimuli in *R. redowskianum*; Table S15: DEGs in *R. redowskianum* uniquely annotated to GO level 3 about sugars, UV light, and microorganisms; Table S16: GO annotations for common up-regulation of *Rhododendron*; Table S17: GO annotations for common down-regulation of *Rhododendron* spp.

## Abbreviations

The following abbreviations are used in this manuscript:

RNA-seq	RNA sequencing
DEGs	differentially expressed genes
DELLA	DELLA domain-containing protein
EDS1	Enhanced Disease Susceptibility 1
UV	Ultraviolet
GO	Gene Ontology
KEGG	Kyoto Encyclopedia of Genes and Genomes
MAPK	Mitogen-Activated Protein Kinase
IPUT1	Inositol Phosphorylceramide Glucuronosyltransferase 1
PGT1	phlorizin synthase
