# Supplementary material for: Adaptive Traits and Molecular Mechanisms of Rhododendron Species in Changbai Mountains’ Alpine Tundra: A Phenotype–Transcriptome Study
Source: Plants (Basel). 2025 Nov 26;14(23):3602. doi: 10.3390/plants14233602 (PMC12694299; doi:10.3390/plants14233602)
Supplement: Supplementary file 1 [file plants-14-03602-s001.zip › Supplementary figure.pdf]

Supplementary file

Adaptive Traits and Molecular Mechanisms of *Rhododendron* Species in Changbai Mountains' Alpine Tundra: A Phenotype-Transcriptome Study

Zhongzan Yang, Jian You, Jiangnan Li, Wei Zhao, Ming Xing, Yuqiao Gong and Xia Chen

National & Local United Engineering Laboratory for Chinese Herbal Medicine Breeding and Cultivation, School of Life Sciences, Jilin University, Changchun, Jilin Province, People's Republic of China
* Correspondence: Xia Chen, Jilin University, Qianjin Avenue 2699, Changchun, Jilin Province, China
E-mail: chenxiajlu@163.com

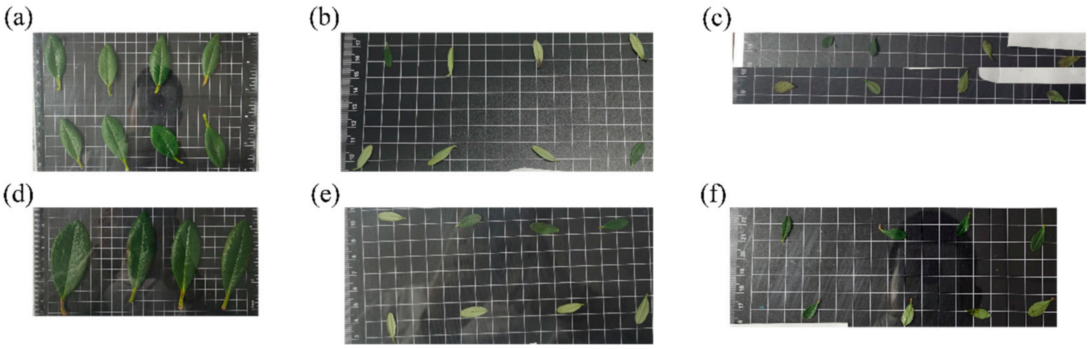

Figure S1 Leaves of three *Rhododendron* species in the tundra and timberline: *R. aureum* (**a**), *R. lapponicum* (**b**), and *R. redowskianum* (**c**) inhabiting the tundra; and *R. aureum* (**d**), *R. lapponicum* (**e**), and *R. redowskianum* (**f**) growing at timberline.

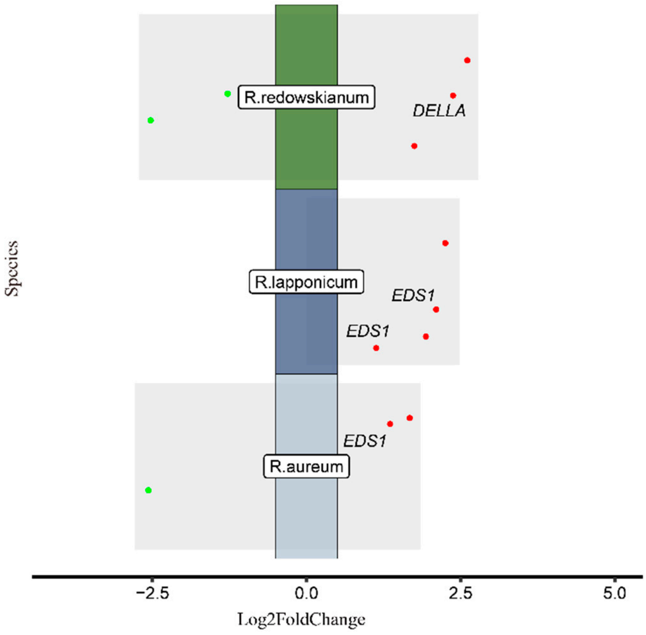

Figure S2 DEGs associated with leaf development annotated in *Rhododendron* spp..

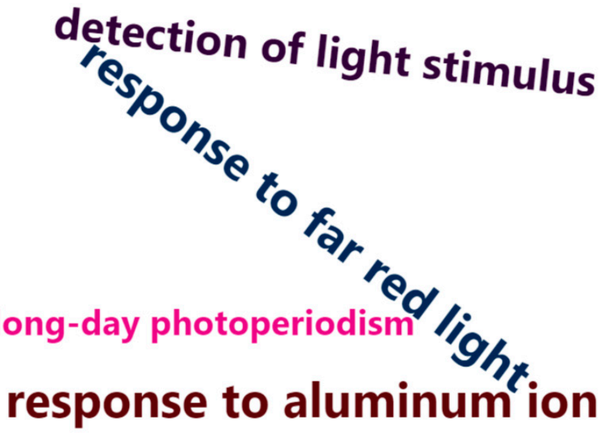

Figure S3 Uniquely annotation to GO level 3 about stimuli in *R. aureum*. Name sizes are randomized, the same below.

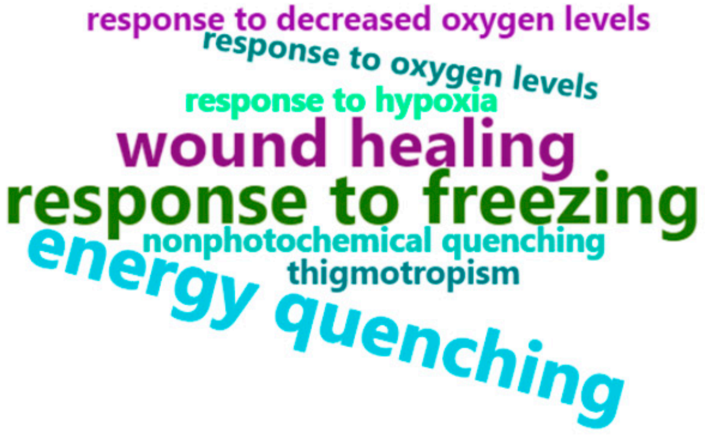

Figure S4 Uniquely annotation to GO level 3 about stimuli in *R. lapponicum*.

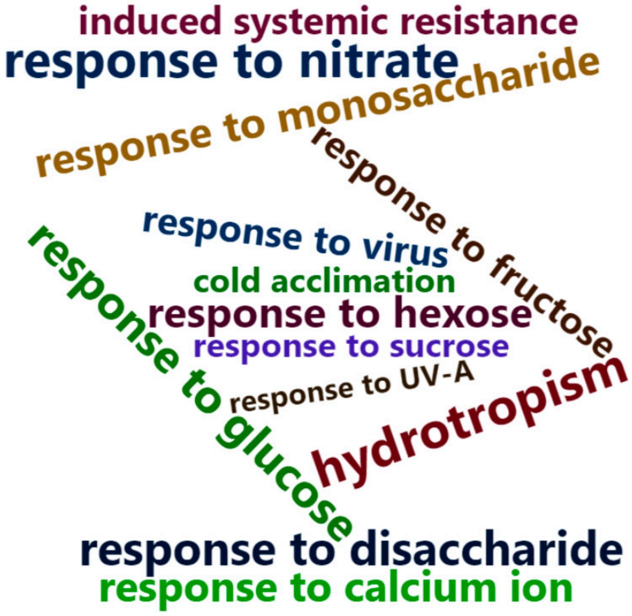

Figure S5 Uniquely annotation to GO level 3 about stimuli in *R. redowskianum*.
